# Supplementary figures and images for: Genetic Diversity of Salmonella Derby from the Poultry Sector in Europe
Source: Pathogens. 2019 Apr 4;8(2):46. doi: 10.3390/pathogens8020046 (PMC6630433; doi:10.3390/pathogens8020046)

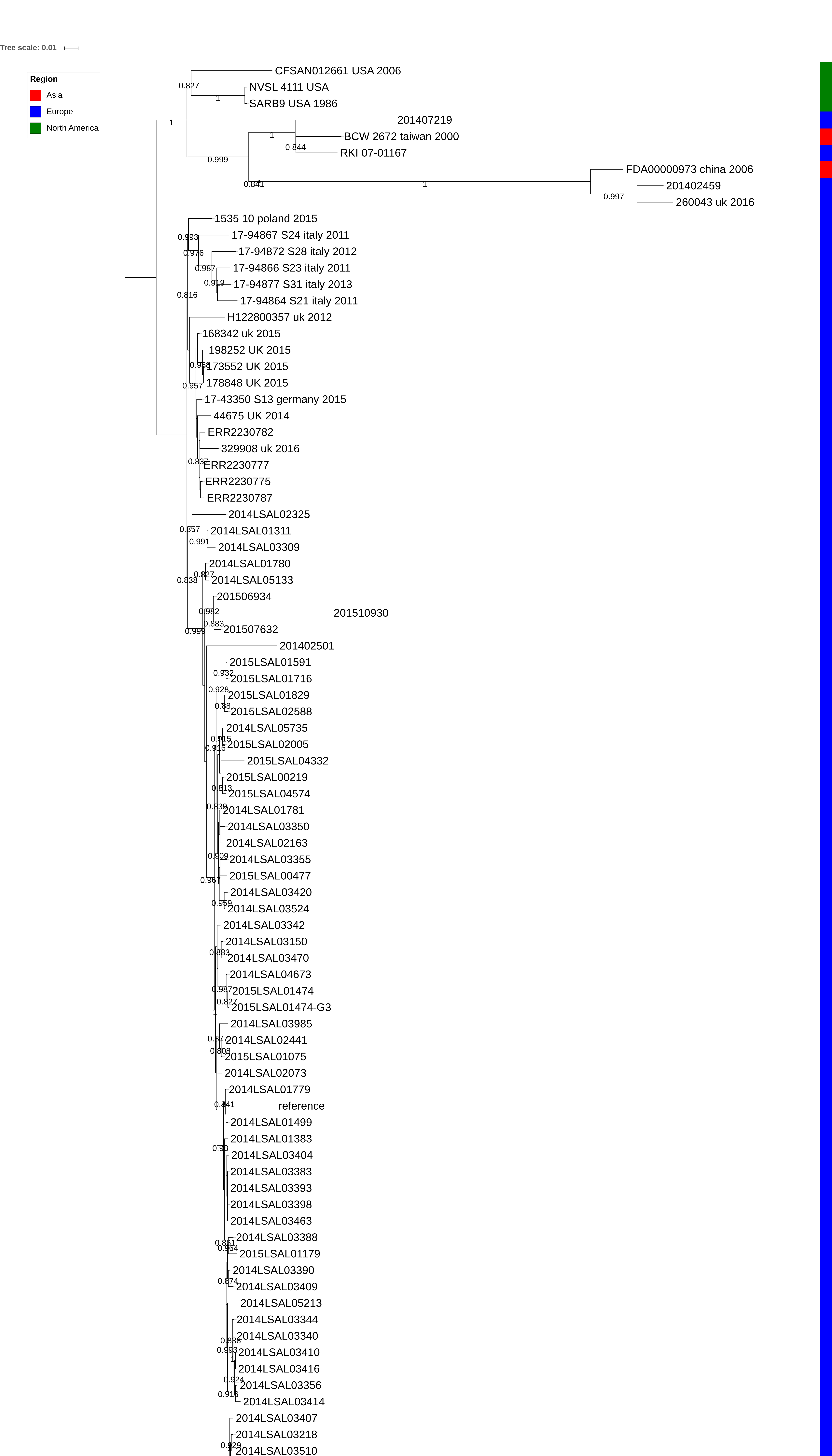

Supplement: Supplementary file 1 [file pathogens-08-00046-s001.zip › S1_Figure.tif]
